# Anterior cutaneous nerve entrapment syndrome with pain present only during Carnett’s sign testing: a case report

**DOI:** 10.1186/s13104-017-2816-1

**Published:** 2017-10-11

**Authors:** Ryutaro Tanizaki, Yousuke Takemura

**Affiliations:** 1 Department of Community Medicine, Nabari, Mie University Graduate School of Medicine, Tsu, Mie Japan; 2 General Medicine, Nabari City Hospital, Nabari, Mie Japan; 3 Department of Family Medicine, Mie University School of Medicine and Graduate School of Medicine, Tsu, Mie Japan

**Keywords:** ACNES, Carnett’s sign, Lidocaine, Abdominal wall pain, Physical examination

## Abstract

**Background:**

The identification of anterior cutaneous nerve entrapment syndrome is often challenging, due to no widely accepted standard guidelines regarding laboratory and imaging tests for the diagnosis of ACNES.

**Case presentation:**

A 77-year-old Japanese man presented with mild lower abdominal pain that had been present for the past 3 years. Physical examination revealed no abdominal pain during palpation, with normal laboratory and imaging testing; therefore, conservative therapy was initiated. However, the abdominal pain continued. Re-examination 16 days later revealed three tender points in accordance with intercostal nerves Th10, Th11, and Th12, with the pain occurring only during Carnett’s sign testing. A cutaneous injection of 1% lidocaine was administered, and the abdominal pain was resolved about 30 min later. Based on these results, anterior cutaneous nerve entrapment syndrome was diagnosed.

**Conclusions:**

It is sometimes hard to diagnose anterior cutaneous nerve entrapment syndrome without testing for Carnett’s sign. If patients present with chronic abdominal pain, clinicians should test for Carnett’s sign even if no pain is elicited during regular abdominal palpation.

## Background

Anterior cutaneous nerve entrapment syndrome (ACNES) is a condition in which chronic or intermittent abdominal wall pain is caused by irritation of the cutaneous nerve roots passing through the abdominal fascia [1]. The incidence of ACNES is still unclear, and may account for 10–30% of patients with chronic abdominal pain in gastroenterological practice [2, 3] and 2% of patients presenting with acute abdominal pain in the emergency department [4]. When ACNES is suspected, cutaneous injection of an anesthetic agent into the painful area is used for both diagnosis and treatment [5]; if pain continues after anesthetic injection, surgery might be required [6, 7].

Carnett’s sign is a clinical examination finding that is useful for confirming whether pain originates from the abdominal viscera or from the abdominal wall. During testing for Carnett’s sign, the investigator identifies the point of maximal abdominal pain by deeply palpating with a finger; the patient is then asked to tense the abdominal muscles while the fingertip is released, followed again by deep palpation. If both stages of the test are painful, the source of the pain is the abdominal wall. In contrast, pain originating from the abdominal organs is associated with just a painful first stage [8]. Although Carnett’s sign is considered useful for diagnosing ACNES [9], identification of ACNES is often challenging due to poor recognition and a lack of widely accepted standard guidelines regarding laboratory and imaging tests for the diagnosis of ACNES. Furthermore, as the degree of pain can vary from mild to severe, if a patient presented with severe pain, ACNES might be misdiagnosed as visceral disease. Herein, we describe a case of ACNES in which the patient presented with no abdominal pain during regular palpation, but experienced abdominal pain when Carnett’s sign was examined.

## Case presentation

A 77-year-old Japanese man presented with chronic mild lower abdominal pain for the past 3 years. The pain had occurred spontaneously and was exacerbated on an empty stomach and during feelings of stress, without any other accompanying symptoms. Despite the chronic abdominal pain, the patient was able to mountain climb as a hobby. The results of abdominal computed tomography and upper gastrointestinal endoscopy done at a local hospital were normal. Rebamipide and lansoprazole had been administered for abdominal pain by a local doctor, but the symptoms did not resolve. The patient had a past medical history of active pulmonary tuberculosis 2 years previously, and polypectomy of a colonic polyp 1 year previously.

Physical examination revealed that the patient’s body temperature was 36.4 °C, blood pressure was 128/60 mmHg, pulse rate was 66 beats/min, respiratory rate was 15/min, and oxygen saturation was 95% (room air). There were no remarkable abnormalities of the head, eyes, ears, nose, mouth, chest, and extremities. There was no tenderness of the abdominal region during palpation, and swab testing, alcohol testing, and skin pinching all produced negative results. Carnett’s sign was not examined at that time. All laboratory investigations showed values within normal range. Thoracic magnetic resonance imaging was performed for suspected thoracic nerve pain, but no abnormalities were detected. After this investigation, conservative therapy was started for unidentified abdominal pain likely originating from a psychiatric etiology.

**Fig. 1:**
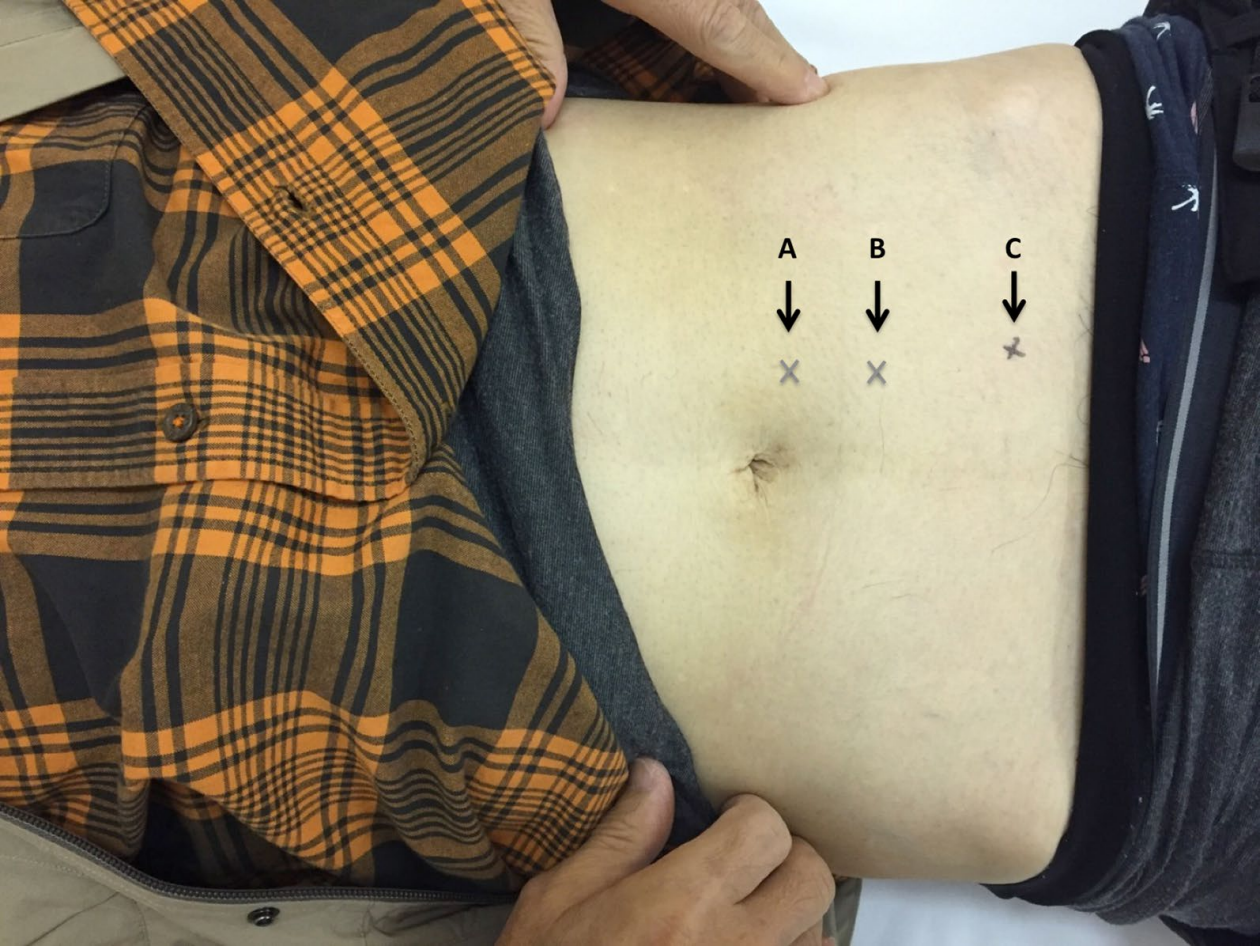
Three tender points ranging within one finger-length along the lower left side of the rectus abdominis muscle

At follow-up examination 7 days later, the symptoms had improved slightly, but the abdominal pain recurred 9 days later. Repeated physical examination of the abdominal region by palpation still revealed no tenderness, but we found three tender points in accordance with intercostal nerves Th10, Th11, and Th12, ranging within one finger-length along the lower left side of the rectus abdominis muscle; the pain was present only when the patient’s abdominal wall was tensed (when Carnett’s sign was examined) (Fig. 1), although local sensory disturbances were not seen. ACNES was suspected, and a diagnostic cutaneous injection of 5 ml of 1% lidocaine was administered by freehand technique without ultrasound (US) guidance at each of the upper two tender points, which had the most marked pain (Fig. 1A, B). Thirty minutes after the injections, the pain had reduced from 10/10 to 2/10 on a pain scale where 0 indicates no pain and 10 represents maximum pain.

One week later, the pain at the sites where 1% lidocaine had been injected was resolved, but the pain remained on the lower left side of the navel (Fig. 1C). Hence, we injected 10 ml of 1% lidocaine into the subfascial space at the remaining painful point under US guidance, and the pain had completely resolved to 0/10 by 30 min after the injection. Due to the above results, ACNES was diagnosed, and there has been no recurrence of symptoms in 4 months after the treatment.

## Discussion

Anterior cutaneous nerve entrapment syndrome is not a fatal disease, but it is hard to diagnose due to its relatively low recognition. Difficulty in diagnosing ACNES could lead to excessive blood tests and imaging studies being conducted, which could cause psychological, physical, and economical burden to patients [10]. Thus, identification of ACNES is important to reduce these burdens. Abdominal wall diseases, including ACNES, should be suspected as a pain source based on appropriate medical interview revealing the existence of infrequent or episodic constant or fluctuating pain, pain intensity related to posture, and pain unrelated to meals or bowel function [4, 6, 11]. In addition, testing for Carnett’s sign is essential for diagnosing ACNES [9, 12]. However, this test is often omitted, as visceral diseases are usually suspected first and abdominal wall diseases may be overlooked as a source of abdominal pain. Diagnosing ACNES is not difficult when Carnett’s sign is examined.

In the present case, the patient experienced chronic lower abdominal pain, but there was no tenderness in the abdominal region during regular examination. Furthermore, the cause of pain had not been found by numerous studies including abdominal computed tomography, upper gastrointestinal endoscopy, and colonoscopy conducted while the pain was present. A local physician had suspected that the abdominal pain had a psychiatric etiology or medically unexplained symptoms during the patient’s first visit, and Carnett’s sign was not initially examined, which might have delayed definitive diagnosis.

Cutaneous injection of a local anesthetic such as 1% lidocaine into the tender points is considered useful for diagnosis as well as treatment of ACNES. However, the long-term effect of 1% lidocaine injection is unclear. Boelens et al. reported that 32.6% of patients who received an injection of 1% lidocaine at tender points became permanently pain-free during their study period, although 51.1% of the patients who received lidocaine injection eventually underwent neurectomy [5]; however, it is unclear whether the injections were administered with US guidance. The lidocaine injection is likely to be more effective when administered in the subfascial space rather than in the subcutaneous space. A freehand injection performed without US guidance may underestimate the treatment effect [13]. In our case, a single injection of 1% lidocaine at each tender point completely relieved the pain during 4 months of follow-up. At the first injection, we administered 1% lidocaine injection by freehand technique without US guidance, which did not completely relieve the pain (remained 2/10 on a pain scale); in contrast, we administered the second 1% lidocaine injection in the subfascial space with US guidance, which resulted in complete pain relief more rapidly than the injections administered using the freehand technique.

A lidocaine injection cannot always provide complete pain relief for patients with abdominal wall pain, but is diagnostic as it can prove that the pain originates from the abdominal wall rather than from visceral disease. In fact, Suleiman et al. suggested that a major goal of lidocaine injection at the tender point is to confirm the pain source [11].

## Conclusions

We experienced a case of ACNES in which the pain was present only during Carnett’s sign testing. If patients present with localized chronic or intermittent abdominal pain with normal laboratory or imaging results, physicians should check for Carnett’s sign even if no abdominal tenderness is identified by regular abdominal palpation. If abdominal wall diseases (including ACNES) are suspected, cutaneous injection of an anesthetic agent such as lidocaine should be administered.

## Abbreviation

ACNES: anterior cutaneous nerve entrapment syndrome
